# Supplementary figures and images for: Chitosan nanoparticles improve the effectivity of miltefosine against Acanthamoeba
Source: PLoS Negl Trop Dis. 2024 Mar 25;18(3):e0011976. doi: 10.1371/journal.pntd.0011976 (PMC10962830; doi:10.1371/journal.pntd.0011976)

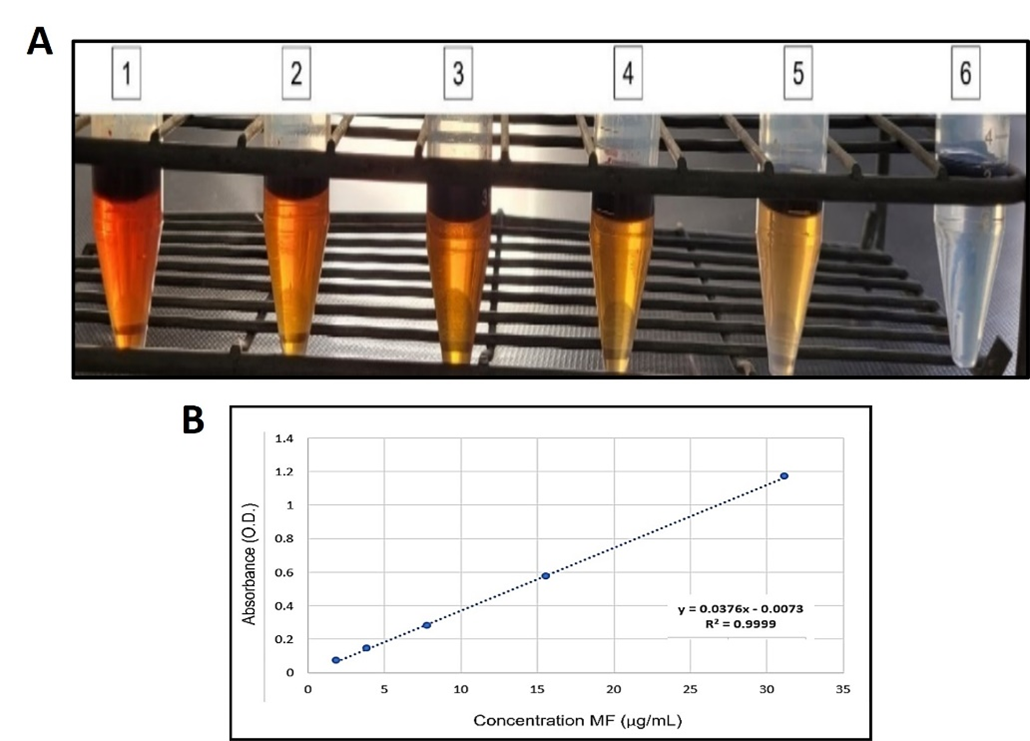

Supplement: S1 Fig — (A) The colored complex results from different concentrations of 0, 2, 4, 8, 16, and 32 μg/mL MF, photographed by the authors (B) the calibration curve of the colorimetric assay. (TIF) [file pntd.0011976.s001.tif]
