# Supplementary material for: Chitosan nanoparticles improve the effectivity of miltefosine against Acanthamoeba
Source: PLoS Negl Trop Dis. 2024 Mar 25;18(3):e0011976. doi: 10.1371/journal.pntd.0011976 (PMC10962830; doi:10.1371/journal.pntd.0011976)
Supplement: Attachment — Submitted filename: Respond to reviewers.docx [file pntd.0011976.s002.docx]

**Dear Prof. Shaden Kamhawi**

**Editor- in Chief,**

**PLOS Neglected Tropical Diseases	26-January-2024**

Regarding your email concerning our manuscript entitled: "Chitosan nanoparticles improve the effectivity of miltefosine against *Acanthamoeba,*" I would like to thank you for your informative email and the reviewers for their valuable and critical comments. Kindly inform you that all the following corrections and changes were done within the manuscript based on reviewers' suggestions. New and increased subjects have been bolded in the manuscript.

We hope the changes listed below meet your requirements.

Overall, I sincerely appreciate your consideration regarding this case and look forward to hearing from you.

Sincerely Yours,

Elham Kazemirad,

Corresponding author, Assistant Professor of Medical Parasitology

Department of Parasitology & Mycology, Tehran University of Medical Sciences,

Tel/Fax: +9821-88951392

[kazemirad@tums.ac.ir](mailto:kazemirad@tums.ac.ir)

[ekazemirad@yahoo.com](mailto:ekazemirad@yahoo.com)

**Reviewer 2:**

**I've completely gone through the manuscript titled "Chitosan nanoparticles improve the effectivity of miltefosine against Acanthamoeba". The authors report the effects of miltefosine loaded chitosan nanoparticles against the two forms of Acanthamoaba namely, cultured trophozoites and cyst form. The cytotoxicity and hemolytic effects of the MF-CS-NPs were also determined on Vero cell line and human RBCs, respectively. This is a well-conceived and well-carried-out study that have yielded valuable data. However, before this paper can be published, a number of flaws must properly be taken care of. The authors should have a native English speaker revise the manuscript before resubmitting it in order to correct the annoying spelling, grammatical, syntactic, and stylistic errors.**

***Response***: Thank you for this comment; the text was edited.

**General and specific comments for the authors**

**Introduction**

**•    The Introduction is not for the authors perform a review about the subject but to clearly show why the study is relevant and how the data obtained will contribute to the management of AK. Most of the references cited in this part are outdated.**

***Response:*** All the references have been rechecked, and we tried to use up-to-date ones. Moreover, the introduction section was revised based on the reviewer's comments.

**•    The authors are advised to pay special emphasis on recent challenges in treatment options of AK.**

***Response***: Thank you for pointing this out. The introduction was edited to emphasize the recent challenges in treating AK.

**Methods**

**•    How the authors confirm the synthesis of NPs**

***Response:*** Thank you for your precise question; the nanoparticle synthesis was confirmed by determining the size and structural properties of NPs. The particle size was determined by three methods, including Dynamic Light Scattering (DLS), Field emission scanning electron microscopy (FE-SEM), and Transmission Electron Microscopy (TEM) (Figs 2-4). Also, the structural properties of MF, CS-NPs, and MF-CS-NPs were demonstrated with FTIR. A high double peak of 2917 and 2850 cm^-1^ was related to CH_2_ stretching in the long hexadecyl chain, indicating MF in nanocarriers (Fig 5).

**•    How the authors standardized the dosing in the experiments**

***Response:*** Thank you for your relevant question; the drug concentration for the experiments was determined and standardized based on our preliminary study and previous reports [1-6].

**•    The authors are advised to perform TEM microscopy of the MF-CS-NPs**

***Response:*** Thank you for your precise comment. Transmission electron microscopy (TEM) was conducted, and the average size was 46.61 nm. The description and figure were added in the M&M, and the result sections (highlighted in green color).

**•    No information is given about how long the isolate of Acanthamoaba used was obtained.**

***Response:*** This isolate was derived from a patient suffering from *Acanthamoeba* keratitis in December 2020, and after determining the genotype, it was kept axenically [7].

The text was edited, and this sentence was added to the M&M (highlighted in yellow color).

**•    The authors should clearly mention the positive and negative control in their experiments**

***Response:*** Chlorhexidine (0.02%) and untreated trophozoites or cysts were used as positive and negative controls in the drug assay, respectively [8]. The 0.02% chlorhexidine as a positive control resulted in 100% death in trophozoite and cyst forms after 24 and 48 hours. The text was edited, and the positive and negative control was added in the M&M and result (highlighted in turquoise color).

•    **How the authors measured the encapsulation efficacy and drug loading content of the MF-CS-NPs**

***Response:***	Thank you for pointing this out. The encapsulation efficacy and drug loading content were determined using the following procedure, which also edited the M&M, highlighted in green.

MF-CS-NPs were prepared from MF (5 mg/mL, 1 mL), TPP (1 mg/ml, 1 mL), and chitosan (2 mg/mL, 5 mL) under constant magnetic stirring at 400 rpm and room temperature. The solution was pelleted by centrifugation at 15,000g for 45 min at 4^º^C, and then the concentration of nonencapsulated MF was determined in the supernatant. Since MF has no UV absorbance, the concentration of MF was measured by a colorimetric assay based on the complexation of zwitterionic MF with anionic ammonium ferric thiocyanate (NH4Fe[NCS]_4_) dye to form a colored complex with ammonium ferric thiocyanate, which can be extracted in organic solvents. This procedure was modified for MF, and 1, 2-dichloroethane was used to extract the brown-red-coloured complex; then, the result was visually and spectrophotometrically assessed at 460 nm. (Fig S1) [9]. The calibration curve was plotted using concentrations of 2, 4, 8, 16, and 32 µg/mL MF based on the complexation of miltefosine with ammonium iron thiocyanate dye (Fig S1). Afterward, the non-trapped MF was estimated in the supernatant medium. Finally, the drug loading of MF in the chitosan nanoparticles was evaluated by determining the difference between the concentration of MF before preparing the nanoparticles and the non-trapped MF. The following equations were used to calculate the encapsulation efficiency (EE%) and loading efficiency (LE%):

$$EE \left( \% \right)=\frac{Total MF-Free MF}{Total MF}\times100$$

$$LE \left( \% \right)=\frac{Total MF-Free MF}{Weight of Nanoparticles}\times100$$

In detail, based on these formulas, LC and EE were calculated as follows:

Free MF: 0.7 mg; MF in the supernatant was calculated by the colorimetric assay.

Total MF: 5mg

Weight of Nanoparticles: 14.25 mg

$$EE=\frac{5mg-0.7mg}{5mg}=86\%$$

$$LE=\frac{5mg-0.7mg}{14.25mg}=30.17\%$$

Fig. S1. The colorimetric assay for miltefosine based on the colored complex formation with ammonium ferric thiocyanate. (A) The colored complex results from different concentrations of 0, 2, 4, 8, 16, and 32 µg/mL MF, photographed by author (B) the calibration curve of the colorimetric assay.

- This statement was edited in the chapter "Evaluation of drug encapsulation" in the M&M, and the figure was added as a supplementary (Fig S1), written in red color.

**•    Penicillin and streptomycin from which source? company, country, and city name**

***Response:*** It was added in the text and highlighted in gray color.

**•    It would be interesting if the authors could perform the experiments in three incubation periods, i.e., 24h, 48 h, and 72h, respectively.**

***Response:*** Thank you for your precise comment. Based on the drug release profile of MF-CS-NPs, miltefosine was released from the nano chitosan after 30 hours. Also, our result showed that the differences between the IC_50_ values of the free MF and the encapsulated drug decreased at 48 h compared to 24 h incubation. After 48 hours, MF-CS-NPS exhibited a slight reduction in cytotoxicity and amoebicidal activity compared to free MF due to the drug release. Based on these results, we omitted the test for 72 hours of incubation.

**•** **In In vitro assay the authors used various concentrations of 9.8, 19.6, 39.2, 78.125, 156.25, 312.5, and 199 625 µg/ml against trophozoites while 156.25, 312.5, 625, 1250, 2500 and 5000 µg/ml for cyst. What was the reason for choosing these concentrations?**

***Response:*** According to the fact that the double-walled cyst is more resistant to drugs than the trophozoite form [10], and also, based on our preliminary study, a greater concentration was selected to determine the MF IC_50_ against the cyst. Likewise, in our result, the IC_50_ value for the cyst form is notably higher than that of the trophozoite form.

**Results**

**The results are well presented. The readers can easily be able to understand what the figures and tables represents.**

**Fig 8 must be removed as it not giving any scientific information**

***Response:*** The image was deleted.

**Discussion**

**The discussion must be written more straightforward. Rather than repeating background information in the first alinea that has already been given in the introduction. The authors should have briefly mentioned the rationale for carrying out the study and give a short version of their findings, and then continue in the subsequent alineas to discuss each finding.**

***Response:*** Thank you for your accurate comment. The discussion section was edited.

**Conclusion**

**Conclusion must be informative. The authors should mentioned their results in this section.**

***Action/Comment:*** The conclusion was edited.

**References**

1.	Walochnik J, Duchêne M, Seifert K, Obwaller A, Hottkowitz T, Wiedermann G, et al. Cytotoxic activities of alkylphosphocholines against clinical isolates of Acanthamoeba spp. Antimicrobial agents and chemotherapy. 2002;46(3):695-701.

2.	Schuster FL, Guglielmo BJ, Visvesvara GS. In‐vitro activity of miltefosine and voriconazole on clinical isolates of free‐living amebas: Balamuthia mandrillaris, Acanthamoeba spp., and Naegleria fowleri. Journal of Eukaryotic Microbiology. 2006;53(2):121-6.

3.	McBride J, Mullen AB, Carter KC, Roberts CW. Differential cytotoxicity of phospholipid analogues to pathogenic Acanthamoeba species and mammalian cells. Journal of Antimicrobial Chemotherapy. 2007;60(3):521-5.

4.	Lukáč M, Mrva M, Garajová M, Mojžišová G, Varinská L, Mojžiš J, et al. Synthesis, self-aggregation and biological properties of alkylphosphocholine and alkylphosphohomocholine derivatives of cetyltrimethylammonium bromide, cetylpyridinium bromide, benzalkonium bromide (C16) and benzethonium chloride. European journal of medicinal chemistry. 2013;66:46-55.

5.	Chao M, Thongseesuksai T, Boonmars T, Laummaunwai P. Investigation of the in vitro cysticidal activity of miltefosine against Acanthamoeba spp. Journal of Parasitic Diseases. 2020;44:491-5.

6.	Mooney R, Williams R. Alkylphosphocholines and quaternary ammonium compounds against Acanthamoeba Keratitis. Journal of Molecular and Clinical Ophthalmology. 2020;2(2).

7.	Soleimani M, Latifi A, Momenaei B, Tayebi F, Mohammadi SS, Ghahvehchian H. Management of refractory Acanthamoeba keratitis, two cases. Parasitol Res. 2021;120(3):1121-4. Epub 2021/01/08. doi: 10.1007/s00436-020-06997-6. PubMed PMID: 33409641.

8.	Sangkanu S, Mitsuwan W, Mahabusarakam W, Jimoh TO, Wilairatana P, Girol AP, et al. Anti-Acanthamoeba synergistic effect of chlorhexidine and Garcinia mangostana extract or α-mangostin against Acanthamoeba triangularis trophozoite and cyst forms. Scientific reports. 2021;11(1):8053.

9.	Dorlo TP, Eggelte TA, de Vries PJ, Beijnen JH. Characterization and identification of suspected counterfeit miltefosine capsules. Analyst. 2012;137(5):1265-74.

10.	Juarez MM, Tartara LI, Cid AG, Real JP, Bermudez JM, Rajal VB, et al. Acanthamoeba in the eye, can the parasite hide even more? Latest developments on the disease. Contact Lens and Anterior Eye. 2018;41(3):245-51.
